# Challenges in the Application of Glyco-Technology to Hepatitis B Virus Therapy and Diagnosis

**DOI:** 10.3390/v13091860

**Published:** 2021-09-17

**Authors:** Tsunenori Ouchida, Shinji Takamatsu, Megumi Maeda, Tatsuya Asuka, Chiharu Morita, Jumpei Kondo, Keiji Ueda, Eiji Miyoshi

**Affiliations:** 1 Department of Molecular Biochemistry and Clinical Investigation, Osaka University Graduate School of Medicine, 1-7 Yamada-oka, Suita 565-0871, Japan; oniyanma1116@gmail.com (T.O.); shinjit@sahs.med.osaka-u.ac.jp (S.T.); maeda.meg.mmm516@gmail.com (M.M.); tasuka25@gmail.com (T.A.); dog.monde0719@gmail.com (C.M.); jumpeko@gmail.com (J.K.); 2 Department of Microbiology, Osaka University Graduate School of Medicine, 2-2 Yamada-oka, Suita 565-0871, Japan; kueda@virus.med.osaka-u.ac.jp

**Keywords:** hepatitis B virus, glycosylation, fucosylation

## Abstract

Hepatitis B virus (HBV) is a major pathogen that causes acute/chronic hepatitis. Continuous HBV infection can lead to the development of hepatocellular carcinoma (HCC). Although several different anti-HBV treatments are available for chronic hepatitis B patients, discontinuing these medications is difficult. Patients with chronic hepatitis B at high risk for HCC therefore require close observation. However, no suitable biomarkers for detecting high-risk groups for HCC exist, except for serum HBV-DNA, but a number of HCC biomarkers are used clinically, such as alpha-fetoprotein (AFP) and protein induced by vitamin K absence-II (PIVKA-II). Glycosylation is an important post-translational protein modification involved in many human pathologic conditions. HBV surface proteins contain various oligosaccharides, and several reports have described their biological functions. Inhibition of HBV glycosylation represents a potential novel anti-HBV therapy. It is thought that glycosylation of hepatocytes/hepatoma cells is also important for HBV infection, as it prevents HBV from infecting cells other than hepatocytes, even if the cells express the HBV receptor. In this review, we summarize considerable research regarding the relationship between HBV and glycosylation as it relates to the development of novel diagnostic tests and therapies for HBV.

## 1. Introduction

Hepatitis B virus (HBV) is a well-known pathogenic virus that causes acute/chronic hepatitis in humans. Worldwide, more than 200 million people suffer from HBV infection [1]. Most patients with chronic hepatitis B acquired the infection via the mother–infant route, called vertical infection, or otherwise horizontal infection such as sexual transmission, drug abuse, and so on, which may involve acute hepatitis caused by a special genotype of HBV (genotype A). Diagnosis of HBV infection is based on the detection of HBsAg/Ab, HBeAg/Ab, HBcrAg, and anti-HBc in blood samples [2]. The clinical status of patients with hepatitis B is evaluated based on these HBV-related Ag/Ab and serum HBV DNA, in addition to liver damage markers such as aspartate aminotransferase (AST) and alanine aminotransferase (ALT) [3]. The standard treatment for chronic hepatitis B is to medicate a nucleotide analogue and interferon [4]. Although nucleotide analogue therapy is very effective, most patients require continuous therapy to suppress HBV replication. Although interferon therapy can result in the complete suppression of HBV replication, the efficacy is relatively low, and the therapy is associated with side effects such as fever and general fatigue.

Sodium taurocholate co-transporting polypeptide (NTCP) was recently identified as a bona fide receptor for HBV and hepatitis delta virus (HDV). NTCP is an integral membrane glycoprotein that functions in the enterohepatic circulation of bile acids [5]. Although numerous reports have suggested that NTCP is an HBV receptor, why the over-expression of NTCP in cells other than hepatocytes or hepatoma cells does not increase the likelihood of HBV infection remains unclear [6]. In the HBV envelope proteins, the preS2 and S regions have a unique oligosaccharide structure. Previous studies using site-directed mutagenesis of potential *N*-glycosylation sites have demonstrated the biological significance of *N*-glycans in HBV envelope proteins [7]. Glycosylation is an important post-translational modification associated with growth factor receptor signaling. Changes in the glycosylation of HBV envelope proteins and/or hepatocyte proteins could therefore affect the risk of HBV infection. Chronic inflammation in the liver has been shown to induce changes in the glycosylation status of serum glycoproteins, which led to the identification of glyco-biomarkers for chronic hepatitis and liver diseases [8]. A recent review on glycosylation and virus infection showed the importance of glycans on viral and host proteins in enveloped virus infection [9]. In cases of enveloped viruses, glycans influence the virus replication cycle, the folding/transport of viral glycoproteins, the release of virus, virus transmission, immune escapes, and virulence/pathogenicity of the virus. Host glycans also affect viral infection in the case of human immunodeficient virus (HIV) and SARS-CoV-2. HBV also belongs to enveloped viruses.

In this review, we summarize previous reports on HBV and glycosylation, as well as our original study. The biological significance of glycosylation, including glycosylation of specific proteins as biomarkers, is discussed in terms of HBV virology and the current state of glycoscience.

## 2. What Is Glycosylation?

Glycosylation is one of the most important post-translational modifications of proteins and lipids. Recent advances in glycobiology and glyco-technology have revealed biological functions of glycosylation in the pathology of various diseases, especially cancers [10,11]. Approximately 50–60% of all proteins are glycosylated, as are most membrane/secreted proteins. Dramatic changes in glycosylation patterns occur at birth and during differentiation, as well as during certain pathologic conditions, such as carcinogenesis and cancer metastasis. Two major types of protein glycosylation have been described: *N*-glycosylation and *O*-glycosylation. In *N*-glycosylation in mammals, *N*-acetylglucosamine (GlcNAc) is attached to the nitrogen atom of an asparagine (Asn) side chain via a β-1 N-linkage within an Asn-X-serine (Ser)/threonine (Thr) motif. In contrast, *O*-glycosylation primarily involves the attachment of GlcNAc/*N*-acetylgalactosamine (GalNAc) to functional hydroxyl groups, most often in Ser and Thr residues. Mucins are *O*-glycosylated proteins characterized by tandem Pro, Ser, and Thr *O*-glycosylated repeats, and the structures of such protein glycosylation change dramatically during malignant transformation. Approximately 1% of human genes are glycosyltransferases involved in the synthesis of oligosaccharides. When a glycosyltransferase acts on the characteristic oligosaccharide structure, it requires a donor substrate. For example, fucosyltransferase uses GDP-fucose to attach fucose to glycans and galactosyltransferase uses UDP-galactose to attach galactose to glycans as each donor substrate. The transporters of GDP-fucose and UDP-galactose are also important for bringing donor substrates from cytosol to Golgi apparatus. The expression of glycosyltransferases is controlled by a variety of transcriptional factors, most of which remain unknown [12].

Glycosylation plays an indirect role in the pathogenesis of a number of human diseases via modification of target glycoproteins. For example, upregulation of growth factor signaling is involved in carcinogenesis. Upregulated growth factor signaling in turn depends on increases in growth factor and/or growth factor receptor levels, as well as post-translational modification of these receptors. Changes in the glycosylation of growth factor receptors can enhance their signaling, ultimately leading to the growth of cancer cells. Growth factor signaling is prolonged by the addition of β1-6 GlcNAc to growth factor receptor *N*-glycans such as EGFR and TGFβ, which is followed by the formation of galectin 3 lattices. The galectin 3 lattices on *N*-glycans inhibit the endocytosis of growth factor receptors after binding of their ligands [13]. Characteristic patterns of serum glycoprotein glycosylation can be used as biomarkers for several diseases [14,15]. Although no driver glyco-genes for the development of cancers and various lifestyle-related diseases have been identified to date, glyco-genes have been implicated as drivers in some congenital disorders of glycosylation [16]. Several human diseases such as cancer and neurological diseases have been linked to disorders of glycosylation [17]. In addition, the glycosylation status of host receptors can play an important role in viral and bacterial infections. Studies of the coronavirus pandemic of 2019 have identified a close association between ABO blood types and susceptibility to infection with the coronavirus [18].

To analyze the glycan structure and its biological function, various technologies have been developed. Lectin microarray is used to understand the comprehensive glycan structure patterns of purified glycoproteins and/or total cells [19]. This method is similar to conventional DNA micro-array for determining gene expression patterns. To confirm a difference of binding to each lectin, lectin blot analysis and/or lectin flow cytometry are used. Furthermore, mass spectrometry analysis is recommended to observe in more detail glycan structures. A combination of mass spectrometry analysis and lectin microarray is an ideal glycan analysis for determining the biological function of glycans and developing glyco-biomarkers [20]. Site-specific analysis of glycoproteins is also important glyco-technology to evaluate glyco-biomarkers [21].

## 3. Biological Significance of the Glycosylation of HBV Surface Proteins

HBV consists of a nucleocapsid protected by a lipid membrane that contains small (S), medium (M), and large (L) surface glycoproteins. These transmembrane proteins are translated from the same open reading frame and have a common carboxy-terminal sequence corresponding to that of the S protein. Early studies by Heermann et al. described HB protein characteristics in detail, including immunogenicity [22,23]. A review by Bruss et al. [24] described various characteristics of HBV, including glycosylation of surface proteins. They reported that a potential *N*-glycosylation site is located at Asn-146 of the S domain [24]. In a more detailed analysis of HBV oligosaccharides [25], Schmitt et al. reported that the M protein has another *N*-glycosylation site at Asn-4, as well as an *O*-glycosylation site at Thr-37 in the preS2 region. Reversed-phase HPLC and matrix-assisted laser desorption/ionization time-of-flight mass spectrometry were employed to analyze in detail the oligosaccharides of HBV surface proteins from patients infected with HBV of different genotypes. These analyses suggest that the *N*- and *O*-glycans of each HBV genotype should have a characteristic glycan structure [25].

**Figure 1:**
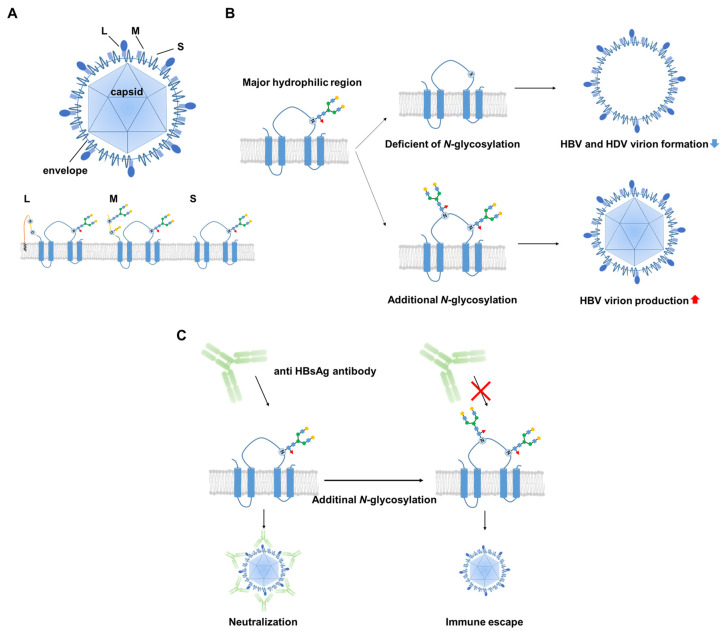
Relationship between HBV and glycosylation. (**A**) Structure of HBV. HBV consists of a nucleocapsid protected by a lipid membrane with small (S), medium (M), and large (L) surface proteins. A potential *N*-glycosylation site is located at Asn-146 of the S domain (blue). M protein has another *N*-glycosylation site at Asn-4 and O-glycosylation site at Thr-37 in the preS2 region (yellow). However, this glycosylation disappears in L proteins. (**B**) The HBV envelope proteins are glycosylated at position N146 in the S domain. Deficiency of *N*-glycosylation on HBV envelope proteins is tolerated for the assembly of subviral HBV particles and the in vitro infectivity of HDV, but is partially inhibitory for the formation of HBV and HDV virions. Additional *N*-glycosylation increases HBV virion production. (**C**) Additional *N*-glycosylation contributes mostly to immune escape and resistance to neutralizing antibody.

Deficiency in *N*-glycosylation of HBV envelope proteins has been shown to have no effect on the assembly of subviral HBV particles, but it partially inhibits the formation of hepatitis D virus (HDV) virions. However, *N*-glycosylation of HBV envelope proteins is not essential for in vitro infectivity of HDV [26]. Based on an analysis of 260 patients with chronic hepatitis B, Yu De-Min et al. reported that *N*-glycosylation site mutations within the major immunogenic region of HBsAg are the primary contributors to immune escape [27]. In vitro experiments involving Huh7 hepatoma cells and Chinese hamster ovary cells transfected with these HBV *N*-glycosylation mutants have revealed that the virion-enveloping and secretion capacity of mutants is greater than that of wild-type HBV [27]. HBV envelope proteins have an *N*-linked glycosylation site at Asn-146, which leads to a nearly 1:1 ratio of glycosylated to non-glycosylated isoforms in the viral envelope. Site-directed mutagenesis studies of this glycosylation site have revealed that it is permissive of envelope protein synthesis/stability and the secretion of subviral particles, but detrimental to HBV virion production. In contrast, hyper-glycosylation of the *N*-linked glycosylation site at Asn-146 has been shown to increase the viral resistance to neutralizing antibodies [7]. These results clearly demonstrate the many biological functions of oligosaccharides on HBV surface proteins in patients with chronic hepatitis B. Figure 1 summarizes HBV glycosylation and its biological functions.

## 4. Is HBV Glycosylation a Useful Target for HBV Therapy and/or Monitoring?

Previous studies using glycosidase inhibitors have demonstrated that failure to process *N*-glycosylation results in the aggregation of HBV particles via impaired protein–protein interactions, ultimately leading to abnormal trafficking [28]. Thus, the glycosidase inhibitor n-butyldeoxynojirimycin could be a novel inhibitor of HBV replication. However, broad-spectrum glycosylation inhibitors could have a variety of side effects. It was recently reported that serine incorporator 5 (SERINC5) is a host intrinsic factor of an anti-human immunodeficiency virus (HIV) [29]. The functional domain of SERINC5 is essential for the inhibition of HBV secretion, but it is not required for inhibition of HIV-1 secretion. SERINC5 increases the non-glycosylation of HBV surface proteins and slightly decreases the levels of HBs protein, leading to decreased HBV secretion. Importantly, SERINC5 co-localizes with the large S HBV protein in the Golgi apparatus. The difference in the inhibitory effects on HBV versus HIV can be attributed to the functional domain of SERINC5. Thus, interfering with the glycosylation of HBV surface antigens is a potent glyco-therapy for HBV.

In a review of clinical data, Qiao et al. reported that an additional *N*-glycosylation mutation in the major hydrophilic region of the HBV S gene is a risk indicator for HCC in patients with chronic hepatitis B [30]. Although the authors analyzed many clinical samples, they did not conduct a detailed investigation of the molecular mechanism, whereby the additional *N*-glycosylation mutation in the HBV S gene increases the risk of HCC. The immune system is known to affect the development of HBV-related liver diseases. In contrast, some papers on PreS deletion mutants and HCC risk have recently been published [31,32], suggesting that glycosylation on PreS lesion is not directly involved in the development of HCC, but rather indirectly involved through immune escapes.

## 5. Glyco-Biomarkers for Hepatitis B-Related Liver Diseases

Block et al. reported the results of a glyco-proteomic analysis to identify glycan biomarkers for HCC, using woodchuck HCC models [33]. For the glyco-proteomic analysis, they employed specific types of lectins that bound to characteristic oligosaccharide structures. Lens culinaris agglutinin (LCA) lectin fractionation of the serum of woodchucks with HCC followed by two-dimensional electrophoresis resulted in the identification of several α1-6-fucosylated proteins. GP73 was identified as a target of α1-6-fucosylation, suggesting fucosylated GP73 as a potential biomarker for human HCC. Using serum samples from a small number of patients with HCC and other chronic liver diseases, the authors also performed LCA lectin precipitation followed by Western blotting of GP73, showing increases in GP73 fucosylation in HCC patients. Additional validation studies have suggested that GP73 is a promising biomarker for patients with chronic liver diseases at high risk of developing HCC [34,35].

Changes in the glycosylation status of specific serum glycoproteins have also been monitored as biomarkers [14]. For example, Mac-2-binding protein glycosylation isomer (M2BPGi) is a biomarker of liver fibrosis, as well as a high potential risk of HCC [36]. Although M2BPGi was originally identified as a liver fibrosis biomarker in patients with hepatitis C, it has also been used as a biomarker of liver fibrosis in other liver diseases, even though the values are typically lower in these cases. With regard to predicting HCC, M2BPGi is a superior biomarker in patients with chronic hepatitis B compared to chronic hepatitis C, as indicated by the results of ROC curve analyses involving 947 treatment-naïve patients with HBV or HCV [37]. Moreover, M2BPGi is both independent of and superior to AFP as a biomarker of HCC risk. Baudi et al. reported that monitoring hepatitis B core-related antigen (HBcrAg) or M2BPGi levels could be suitable for evaluating therapeutic effects and clinical outcomes in terms of prevention of HCC occurrence in patients with hepatitis B, suggesting that they are appropriate surrogate biomarkers for predicting hepatitis B disease progression [38].

Agalactosylation of IgG-Fc in patients with chronic hepatitis B is highly associated with histologically diagnosed liver damage but can be reversed by anti-viral therapy [39]. Agalactosylation of IgG-Fc appears to be associated with chronic inflammation, as well as rheumatoid arthritis and inflammatory bowel diseases. In contrast, Cheng-Husm et al. reported that high levels of fucosyl-agalactosyl IgG in the serum at treatment endpoint would be a favorable response to long-term nucleoside analogue therapy in patients with HBe Ag-positive chronic hepatitis B [39]. Although a number of useful biomarkers [40] for HBV-associated diseases have been reported, such as HBV DNA, HBs Ag/Ab, and HBe Ag/Ab, HBV glyco-biomarkers could provide information regarding the progression of HBV-related liver diseases from a different perspective (Table 1).

## 6. NTCP Glycosylation

NTCP, which contains two *N*-glycans, was recently identified as the bona fide receptor for HBV. *N*-glycosylation is important for the biological function of NTCP. Appelman et al. reported that NTCP in which the amino acid sequence of the *N*-glycosylation site was mutated via site-directed mutagenesis was glycosylation-deficient and failed to support HBV infection due to the effects on trafficking and stability [40], and *N*-glycosylation-deficient NTCP did not localize at the plasma membrane. It has been reported that the glycosylation pattern of NTCP is development-dependent, leading to the localization of NCTP on the surface of hepatocytes [41]. The authors of that study investigated changes in NTCP molecular weight using immunoblotting and hepatocytes isolated from several infant donor livers, and changes in the localization of NTCP were examined by immunostaining.

Other studies have reported that E-cadherin interacts with glycosylated NTCP to promote the entry of HBV into human hepatoma cells [42]. The authors of that study showed that knockdown of E-cadherin significantly reduced infection by HBV particles and the entry of HBV pseudoparticles in infected liver cells and cell lines. Glycosylated NTCP localizes to the plasma membrane via interaction with E-cadherin, which increases the interaction with the preS1 domain of large HBV surface antigens. Thus, glycosylation of NTCP biologically affects several HBV infection processes directly and/or indirectly. A recent study reported that forming a complex of NTCP and EGF receptor is important for HBV internalization [43]. Because EGFR is a glycoprotein with 11 *N*-glycosylation sites, it is of future interest whether or not glycosylation status is involved in the interaction with NCTP.

## 7. HBV Infection and Cell Surface Glycosylation

Although NTCP functions as a cell surface receptor to facilitate the entry of HBV into hepatocytes, HBV does not infect other types of cells, even if they express NTCP. It has been suggested that post-translational modifications of NTCP and/or the co-receptor for NTCP are required for HBV entry into hepatocytes. Previously, we investigated the relationship between cellular glycosylation status and infectivity using pseudo-HBV particles known as bio-nanocapsules (BNCs) [44]. Our experiments examining BNC entry into various HCC cell lines using glycosylation remodeling indicated that core-fucosylation should be an important glycosylation event for enabling the HBV infection of hepatoma cells via HBV receptor-mediated endocytosis. The synthesis of core-fucose is catalyzed by α1-6-fucosyltransferase (Fut8). Markedly increased expression of Fut8 has been observed in HB611 cells, established from Huh6 cells transfected with tandem repeat HBV genome DNA [45]. BNC entry into HB611 cells have been shown to be markedly increased compared to entry into parental Huh6 cells. In addition, over-expression of Fut8 leads to increased BNC entry into Huh6 cells. These data suggest that core-fucose should be a key glycosylation for HBV entry. Analyses of clinically derived HCC tissues has indicated that Fut8 expression could be upregulated in cancer lesions compared to surrounding non-cancerous tissues [46].

An important question that arises then is why is core-fucosylation important for HBV entry? When BNC attachment/entry into Huh6 and HB611 cells was investigated at 4 °C, no changes in Cy3-labeled BNC distribution were observed. In contrast, confocal microscopy images showed a dramatic difference in BNC distribution between HB611 and Huh6 cells when the experiment was conducted at 37 °C. BNC signals were observed on both the cell surface and intracellular portion in HB611 cells, but the signals were limited only on the surface of Huh6 cells [44]. These data suggest that increased BNC incorporation in HB611 cells depends on increased BNC endocytosis via HBV receptors and that this endocytosis is affected by core-fucosylation of the HBV receptor and/or co-receptors. The involvement of core-fucosylation in endocytosis has also been reported with transferrin receptor and Toll-like receptor 4 [47].

**Figure 2:**
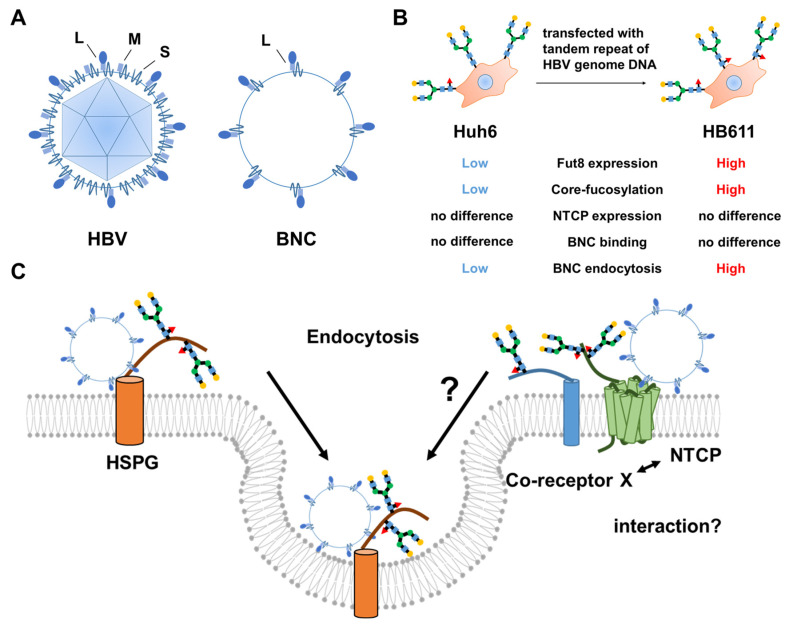
HBV infection and core-fucosylation. (**A**) Structure of BNCs. BNCs consist of only an envelope with large S proteins. Large S proteins bind to NTCP, a cell surface receptor for HBV entry into hepatocytes, through the preS1 region. (**B**) Difference between Huh6 and HB611 cells. HB611 cells were established from Huh6 cells transfected with tandem repeat HBV genome DNA. There were no differences in NTCP expression or BNC binding between Huh6 and HB611 cells. However, HB611 cells were highly core-fucosylated, and BNC endocytosis into HB611 cells was increased. (**C**) Summary of our results and speculation. BNC/HBV internalization into hepatoma cells occurs in a core-fucose-dependent manner. However, it remains unknown which glycans are important in NTCP and/or a putative co-receptor X.

In comparative immunoprecipitation analyses of NTCP between HB611 cells and Fut8-knockdown HB611 cells followed by mass spectrometry, we identified myosin hyper 9 (MYH9) as a key molecule in the NTCP-mediated entry of BNCs into hepatoma cells. Although there was no difference in the NTCP expression level between HB611 and Huh6 cells, the levels of NTCP-associated MYH9 were increased in HB611 cells. As MYH9 is not a glycoprotein, core-fucosylation of NTCP and/or NTCP-associated glycoproteins on the cell surface appears to be important for the entry of BNCs into these cells. The working hypothesis describing the role of core-fucosylation in HBV infection is shown in Figure 2.

## 8. Closing Remarks

Numerous studies have examined the role of glycosylation in the process of HBV infection. Most papers published to date indicate that glycosylation of HBV surface antigens plays an important role in viral sorting on the cell surface and also in virus secretion. In contrast, our own research using pseudo-HBV particles (i.e., BNCs) demonstrated that fucosylation on the surface of hepatoma cells is critical for HBV entry. Our preliminary data suggest that inhibition of core-fucosylation suppresses HBV entry into NTCP-expressing HepG2 cells (data not shown). Although the mechanism of this suppression might not be consistent with our previous study on the molecular mechanisms underlying BNC entry, our data suggest that core-fucose could be a novel target for HBV therapy. The most important issues in glyco-therapy for HBV are how to identify target proteins of core-fucose and/or other key glycosylation. Broad glycosylation inhibitors would be unsuccessful because of their side effects. An ideal glyco-therapy might be to develop a next-generation antibody, which recognize both the characteristic glycan structures and peptides involved in HBV infection and replication. Moreover, combination/modulations of glyco-technology and conventional therapy could be a bona fide therapy. For example, the modulation of IgG glycosylation is used in clinical therapy [48]. Natural products or some small molecules, which mildly alter glycosylation status, might enhance anti-HBV drugs, leading to a novel therapy.

## Figures and Tables

**Table 1:** Glyco-biomarkers for hepatitis B-related liver diseases. M2BP-Gi is Mac-2 binding protein glycosylation isomer recognized by WFA lectin. WFA binds to LacdiNAc structures (GalNAc-GlcNAc). IgG, immunoglobulin G. DSGG, fucosyl GM1, and Gb2, glycolipids. Yellow square, GalNAc; blue square, GlcNAc; yellow circle, galactose; blue circle, glucose; green circle, mannose; red triangle, fucose; pink diamond, sialic acid.

Glyco-Biomarkers	Glycan Changes	Relationship with HBV	Monosaccharides
M2BP-Gi	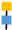 Increase LacdiNAc	appropriate surrogative biomarkers for predicting disease progression of hepatitis B [Baudi et al., 2020]	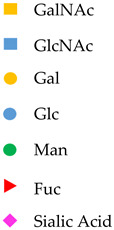
IgG	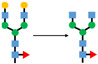 Agalactosylation	Highly associated with histological liver damage and reversed by anti-viral therapy [Ho et al., 2017] High levels of serum fucosyl-agalactosyl IgG at the treatment endpoint is favorable response to long-term nucleoside analogue therapy in patients with chronic hepatitis B. [Ho et al., 2017]	
Anti glycan IgG	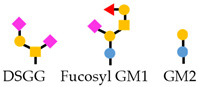	Serum concentrations of anti-DSGG, anti-fucosyl GM1 and anti-Gb2 were significantly higher in patients with HCC than in chronic HBV infection individuals not in chronic HCV infection patients. [Wu et al., 2012]	

## Data Availability

Not applicable.
